# Predictors of heart and lung dose in left-sided breast cancer treated with VMAT relative to 3D-CRT: A retrospective study

**DOI:** 10.1371/journal.pone.0252552

**Published:** 2021-06-09

**Authors:** Zheng Kang, Sijia Chen, Liwan Shi, Yipeng He, Xiang Gao

**Affiliations:** Department of Radiation Oncology, The First Affiliated Hospital of Xiamen University, Xiamen, Fujian Province, China; West China hospital, Sichuan University, CHINA

## Abstract

**Background:**

Before generating radiotherapy plans for breast cancer patients, the choice of plan techniques (three-dimensional conformal radiation therapy (3D-CRT) and volumetric modulated arc therapy (VMAT)) should be made. This study investigated the performance of two geometric indices in aiding the choice of 3D-CRT and VMAT plans in women undergoing left-sided whole breast radiotherapy.

**Materials and methods:**

119 patients, previously treated with left-sided breast radiotherapy (61 3D-CRT treatments and 58 VMAT treatments) from a single institution, were retrospectively studied. Two geometric indices, which were cardiac junction (CJ) index and pulmonary junction (PJ) index, were defined and the relationship between these indices and dose of organs at risk (OARs) were evaluated. Two-tailed Student’s t-test was performed to compare patient characteristics between 3D-CRT and VMAT. Linear regressions were calculated to investigate the association between geometric indices and absorbed dose of heart and left lung, including mean dose of heart (MHD), V_5_, V_30_ of heart, and mean dose of left lung (MLLD), V_5_, V_10_, V_20_, V_30_, V_40_ of left lung.

**Results:**

The CJ index was strongly correlated with the MHD in 3D-CRT group and VMAT group. The linear regression formulas were MHD = 4826.59 ×CJ Index+310.48 (R = 0.857, F = 163.77, P = 0.000) in 3D-CRT plans and MHD = 1789.29×CJ Index+437.50 (R = 0.45, F = 14.23, P = 0.000) in VMAT plans. The intersection of the two formulas was CJ index = 4.2% and MHD = 512.33 cGy. The PJ index demonstrated a strongly positive correlation with MLLD in 3D-CRT group and VMAT group as well. The linear regression formulas were MLLD = 2879.54×PJ Index+999.79 (R = 0.697, F = 55.86, P = 0.000) in 3D-CRT plans and MLLD = 1411.79×PJ Index+1091.88 (R = 0.676, F = 47.11, P = 0.000) in VMAT plans, the intersection of the two formulas was PJ index = 6.3% and MLLD = 1180.46 cGy.

**Conclusions:**

CJ index and PJ index could be used as a practical tool to select 3D-CRT or VMAT before generating plans. We recommend that VMAT plan is preferable when CJ index is greater than 4.2% and/or PJ index is greater than 14.6%, while 3D-CRT plan is the first choice in the opposite.

## Introduction

Post mastectomy radiation therapy (PMRT), which significantly improves local tumor control and increases 5-year overall survival for breast cancer patients, is an effective and well-established adjuvant treatment for breast cancer patients with modified radical mastectomy [1–5].

However, the heart and lungs are routinely exposed to incidental ionizing radiation during adjuvant radiotherapy of breast cancer. It can result in increasing cardio-toxicity and cardiovascular mortality especially in left sided breast cancer patients [6]. Radiation induced heart disease generally occurs when patient follow-up is over 10 years even with modern therapy [7]. Clinical study had shown that rates of major coronary events increased linearly with the mean dose to the heart by 7.4% per Gy, the increase started within the first 5 years after radiotherapy and continued at least 20 years [8]. While another research demonstrated that the cumulative acute coronary event rate increased by 16.5% per Gy [9]. Except for cardio-toxicity, radiation related lung toxicity is also a concern in left-sided breast radiotherapy. Studies suggested that the incidence of symptomatic radiation pneumonitis could range from 3.7% to almost 20% in different studies [10, 11]. Grantzau et al. conducted a research indicated that the risk of second non-breast cancer after radiotherapy of the breast cancer patients, including the lung, esophagus, thyroid and connective tissues progressively increased over time, peaking at 10–15 years following breast cancer diagnosis [12].

Conventionally, the three-dimensional conformal radiation therapy has been widely applied for breast cancer. Due to the concave shape of the thoracic wall, a novel plan technique known as the volumetric modulated arc therapy has also been extensively utilized in clinic recently. Researches indicated that VMAT plans spared the OARs from high-dose volume at the cost of increasing their low-dose volume [13–15], especially in patients with axillary and supraclavicular lymph node areas. In the ipsilateral lung, the VMAT plans demonstrated lower V_20_, V_30_, while higher V_5_, V_10_ compared to 3D-CRT plans [15]. In the heart, the VMAT plans had lower V_5_, V_20_ and V_30_ than the 3D-CRT plans [14]. The low dose bath exposure of healthy tissue raised concerns about late onset secondary cancer [16] or undesirable acute side effects, such as the radiation-induced nausea and vomiting (RINV) [17].

In clinical practice, it is usually time-consuming to create radiotherapy plans. For left-sided breast cancer, we usually do not have the basis to decide whether to choose 3D-CRT or VMAT plan before making treatment plan. To date, few studies [18] have evaluated the role of specific geometric indices in predicting treatment technique selection. In this study, we propose two novel and practical geometric indices related to the dose distribution in the heart and left lung of women receiving 3D-CRT or VMAT left-sided whole breast radiotherapy. Our hypothesis was that these geometric indices could be used as applicable tool for clinical selection of 3D-CRT plan and VMAT plan before actual planning.

## Materials and methods

### Patient population

A retrospective review study, approved by the Ethics Committee of the First Affiliated Hospital of Xiamen University and performed in accordance with the Declaration of Helsinki, was performed to quantitatively assess 119 consecutive women with left-sided breast cancer. The analysis was performed with data extracted from the dose-volume-histogram (DVH) of the treatment plan. The inclusion criteria included: (1) the patients were all female; (2) the primary lesions were left breast; (3) postoperative RT initiated after completion of chemotherapy for patients receiving adjuvant chemotherapy; (4) treatment with 3D-CRT or VMAT; (5) no previous irradiation of the breast; (6) only patients with 6MV beam were enrolled. And informed consent of all patients was obtained. All data were fully anonymized before we accessed them. All patients were treated with whole breast RT after modified radical mastectomy at our institution from 2019 to 2020 using Varian TrueBeam linear accelerator (Varian Medical Systems, Palo Alto, CA). The prescription for the whole breast RT was 50.4 Gy in 28 fractions (1.8 Gy/fraction).

### Contouring and treatment planning

All patients received computer tomography (CT) (GE Lightspeed 16, GE HealthCare) scan. CT images were acquired with patients lying on a breast-board in supine position, at a 0.5 mm slice thickness. Clinical target volume (CTV) was delineated in accordance with the Radiation Therapy Oncology Group (RTOG) guidelines, including the whole ipsilateral chest wall and lymph node region around collar bone, using the Eclipse treatment planning system (Eclipse 11.0, Varian Medical Systems, Palo Alto, CA, USA). The planning target volume (PTV) was generated by expanding the CTV with a 5 mm margin in all directions, subsequently retracted 0.1cm and 0.5 cm from the body surface in the chest wall section and supraclavicular section, respectively.

All treatment plans were generated using 6MV photon beam in Eclipse V.11.0 (Varian, USA). The VMAT plans with 6 partial arcs were generated, while the 3D-CRT plans with 5 field-in-field fields including two tangential fields in the chest wall region were obtained. The arc arrangement was consistent in VMAT plan and beam arrangement in 3D-CRT plan was consistent. Tissue equivalent compensator was placed over the surface of the chest wall to ensure sufficient target coverage near the chest wall surface with thickness of 1cm for the VMAT group, and 0.3cm for the 3D-CRT group. Dose metrics were calculated based on the cumulative DVH, including V_5_, V_10_, V_20_, V_30_, V_40_, MLLD of the left lung, and V_5_, V_30_, MHD of the heart. All plans were reviewed by two physicists and being approved by a radiation oncologist.

### PTV tangential field and cardiac and pulmonary junctions

**Fig 1:**
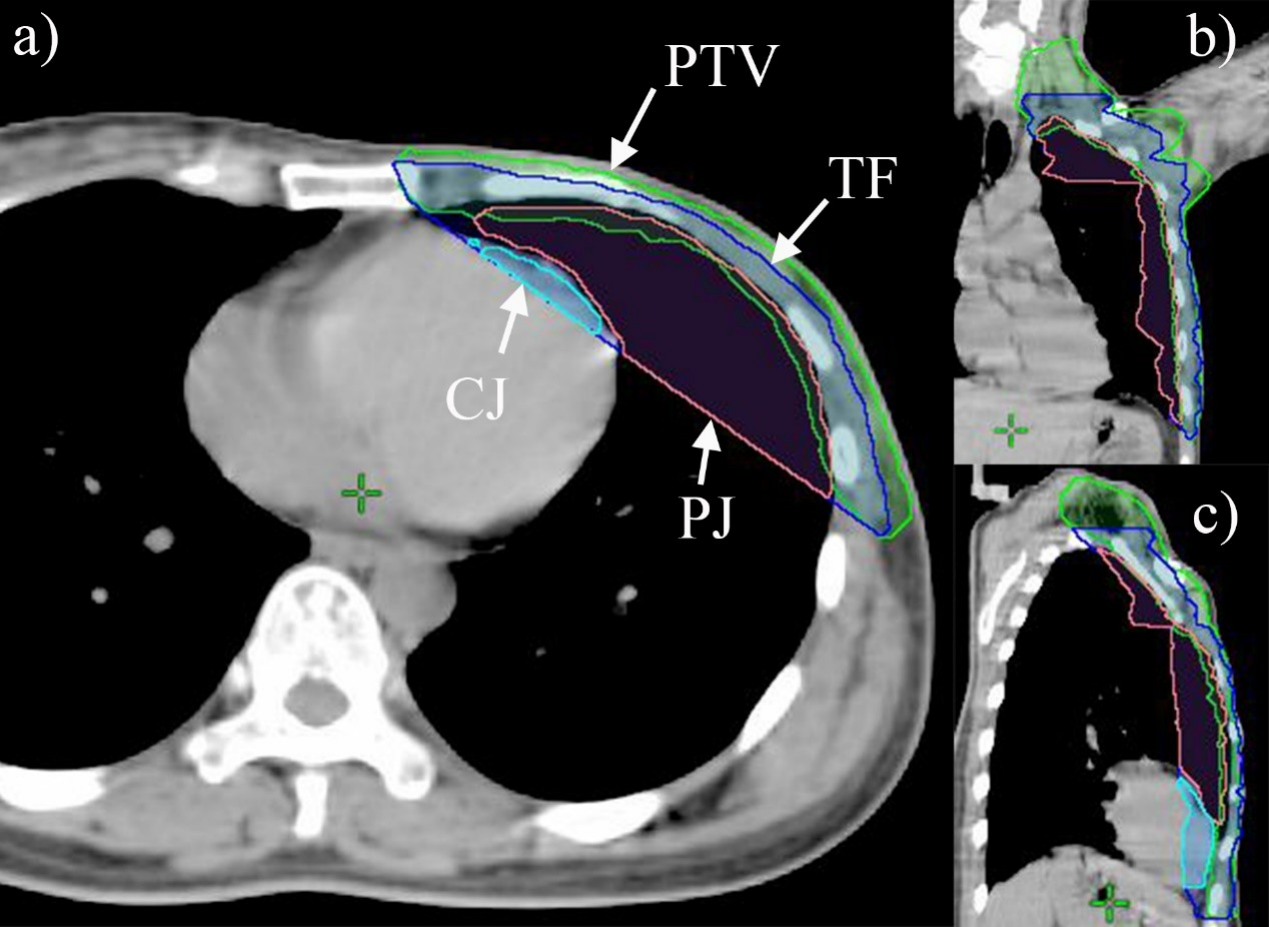
Geometric metrics displayed in axial (a), coronal (b) and sagittal (c) slices. The green, blue, cyan and pink contours were PTV, TF, CJ and PJ, respectively.

The PTV tangential field (TF) was defined as the area between the posterior tangent of the PTV and the chest wall from the tip of left lung to the bottom of PTV (Fig 1). In this work, we drew a TF layer every two CT slices, and utilized the interpolate tool in Eclipse V.11.0 to form the overall contour of TF. The cardiac junction (CJ) was determined as the region where the heart and TF intersect, as illustrated in Fig 1. The pulmonary junction (PJ) was defined as the region where the left lung and TF intersect. Then, the volume of CJ and PJ was measured to calculate CJ and PJ indices. The CJ index is calculated as the ratio of CJ volume to heart volume, and the PJ index is calculated as the ratio of PJ volume to left lung volume. We believed that CJ index and PJ index could be used to evaluate the degree of radiation exposure of heart and left lung. Therefore, this study evaluated the relationship between CJ index and absorbed dose of heart, and PJ index and absorbed dose of left lung.

### Statistical analysis

Descriptive statistics were summarized for all doses and geometric metrics. Two-tailed Student’s t-test was performed to compare patient characteristics between 3D-CRT and VMAT. Linear regressions were calculated to investigate the association between geometric indices and absorbed dose of heart and left lung, including MHD, V_5_, V_30_ of heart, and MLLD, V_5_, V_10_, V_20_, V_30_, V_40_ of left lung. Statistical significance was defined at the *p* = 0.05 significance level, and the data was presented as mean ±SD. The IBM SPSS Statistics V22 software was used for all statistical analysis.

## Results

One hundred and nineteen patients, aged 26 to 69 years old, with modified radical mastectomy were included in this study. Clinical and treatment characteristics of each cohort and their geometric parameters were summarized in Table 1. There were no significant differences in age, heart volume, CJ volume and left lung volume between 3D-CRT group and VMAT group. However, significant difference was noted between 3D-CRT group and VMAT group with respect to PJ volume. The CJ volume and PJ volume in each cohort were highly variable. The CJ volume was 30.63±20.55 cc in 3D-CRT group compared to 37.78±22.34 cc in VMAT group. The PJ volume in 3D-CRT group and VMAT group were 146.26±53.50 cc and 194.82±61.16 respectively. Compared with 3D-CRT, the PJ volume of VMAT was 48.56 cc larger. CJ index of 3D-CRT group and VMAT group were0.058±0.037 and 0.069±0.033, respectively. PJ index of 3D-CRT group and VMAT group were 0.146±0.032 and 0.188±0.043, respectively.

**Table 1:** Characteristics of patients treated with 3D-CRT and VMAT.

Characteristics	3D-CRT N = 61, mean±SD	VMAT N = 58, mean±SD	*P-Value*
Age (year)	45.6.0±10.5	48.2±9.3	0.158
Heart Volume (cc)	521.36±92.75	535.63±76.89	0.364
T stage			
1	15	11	
2	32	32	
3	13	12	
4	1	3	
N stage			
0	6	2	
1	36	26	
2	12	19	
3	7	11	
PTV Volume (cc)	811.36±316.85	794.94±272.44	0.763
Heart Volume (cc)	521.36±92.75	535.63±76.89	0.364
CJ Volume (cc)	30.63±20.55	37.78±22.34	0.099
CJ Index (%)	5.8±3.7	6.9±3.3	0.099
Left Lung Volume (cc)	996.90±268.54	1041.07±227.44	0.336
PJ Volume (cc)	146.26±53.50	194.82±61.16	0.000
PJ Index (%)	14.6±3.2	18.8±4.3	0.000

The MHD was similar in the 3D-CRT and VMAT cohort (*p* = 0.368), which were 589.43±209.82 cGy and 560.19±132.38 cGy, respectively (Table 2). The V_5_ of heart was dramatically larger in the VMAT plans than that in 3D-CRT plans, suggesting 3D-CRT could reduce low-dose radiation to the heart, relative to the VMAT technique. Nevertheless, in 3D-CRT and VMAT plans, V30 of heart showed an opposite trend, and the V_30_ of heart in 3D-CRT plan increased by 5% (Table 2).

**Table 2:** Dosimetric parameters of OARs.

Absorbed dose	3D-CRT n = 61, mean±SD	VMAT n = 58, mean±SD	*p*-Value
Heart			
Mean dose (cGy)	589.43±209.82	560.19±132.38	0.368
V_5_ (%)	16.46±6.25	28.17±8.02	<0.01
V_30_ (%)	7.65±3.87	2.35±1.65	<0.01
Left lung			
Mean dose (cGy)	1420.31±133.65	1356.09±89.93	<0.01
V_5_ (%)	45.34±3.80	56.17±3.73	<0.01
V_10_ (%)	32.92±3.09	39.98±3.55	<0.01
V_20_ (%)	26.56±2.90	25.31±2.43	0.013
V_30_ (%)	23.58±2.88	16.70±1.76	<0.01
V_40_ (%)	19.56±2.80	9.68±1.60	<0.01
Right lung			
Mean dose (cGy)	78.25±27.80	439.50±84.97	<0.01
V_5_ (%)	2.25±1.91	29.85±8.85	<0.01
V_20_ (%)	0.01±0.05	0.55±0.60	<0.01
Right breast			
Mean dose (cGy)	75.32±46.44	450.96±92.33	<0.01
V_5_ (%)	0.77±1.81	28.25±10.64	<0.01
HI (%)	0.16±0.03	0.10±0.17	<0.01

**Fig 2:**
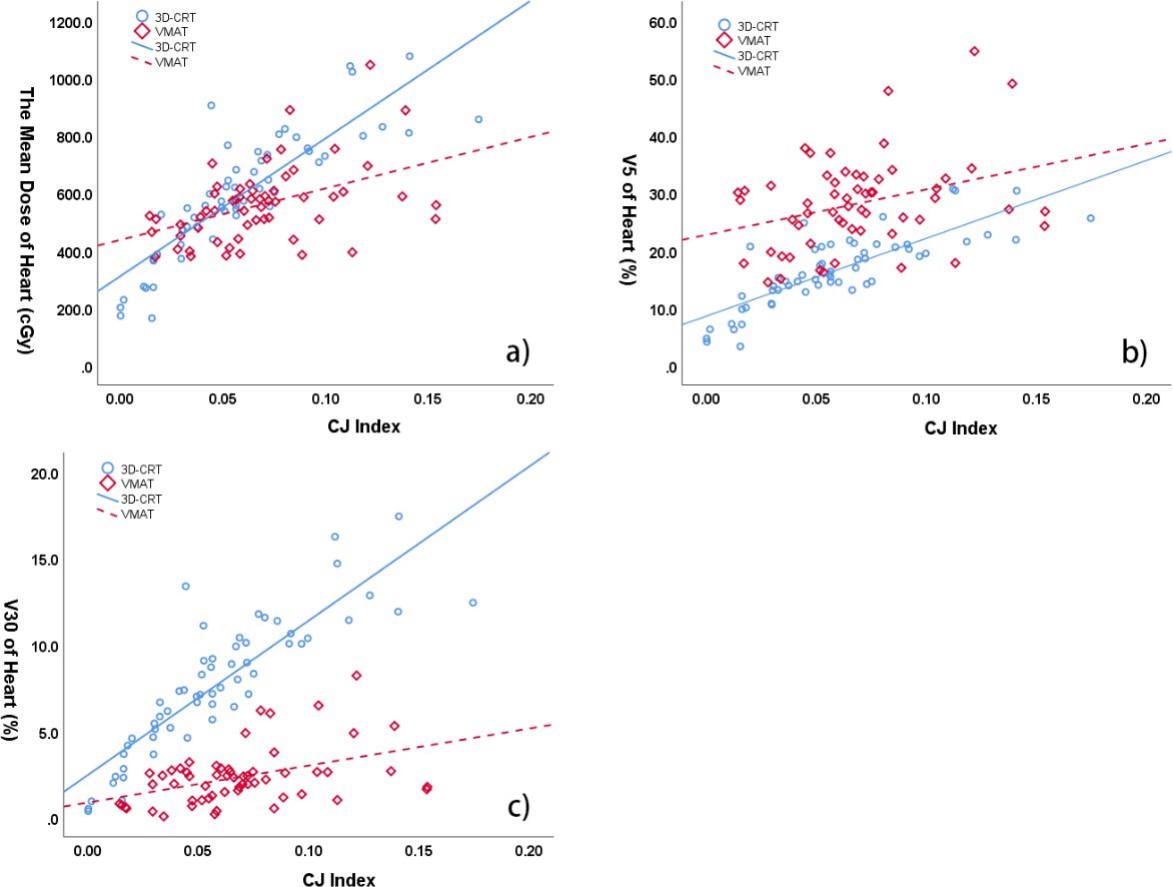
Correlation between CJ index and dose metrics of heart in 3D-CRT and VMAT. a) CJ index and the mean dose of heart (r = 0.857 for 3D-CRT, *p* < 0.01; r = 0.45 for VMAT, *p* < 0.01); b) CJ index and V5 of heart (r = 0.814 for 3D-CRT, *p* < 0.01; r = 0.328 for VMAT, *p* < 0.01); c) CJ index and V30 of heart (r = 0.869 for 3D-CRT, *p* < 0.01; r = 0.431 for VMAT, *p* < 0.01).

The CJ index was strongly correlated with the MHD (r = 0.857, *p* < 0.01), V_5_ of heart (r = 0.814, *p* < 0.01) and V_30_ of heart (r = 0.869, *p* < 0.01) in 3D-CRT group (Fig 2). There was positive correlation between CJ index and the MHD(r = 0.45, *p* < 0.01), V_5_ of heart (r = 0.328, *p* < 0.01), as well as V_30_ of heart (r = 0.431, *p* < 0.01) in VMAT group (Fig 2).

The MLLD was 1420.31±133.65 cGy in 3D-CRT group and 1356.09±89.93 cGy in VMAT group (Table 2), and the difference between the two groups was statistically significant. As expected, 3D-CRT produced less low-dose radiation (V_5_ and V_10_) to the left lung than VMAT, and more high-dose radiation to OAR (V_20_, V_30_ and V_40_) than VMAT (Table 2).

**Fig 3:**
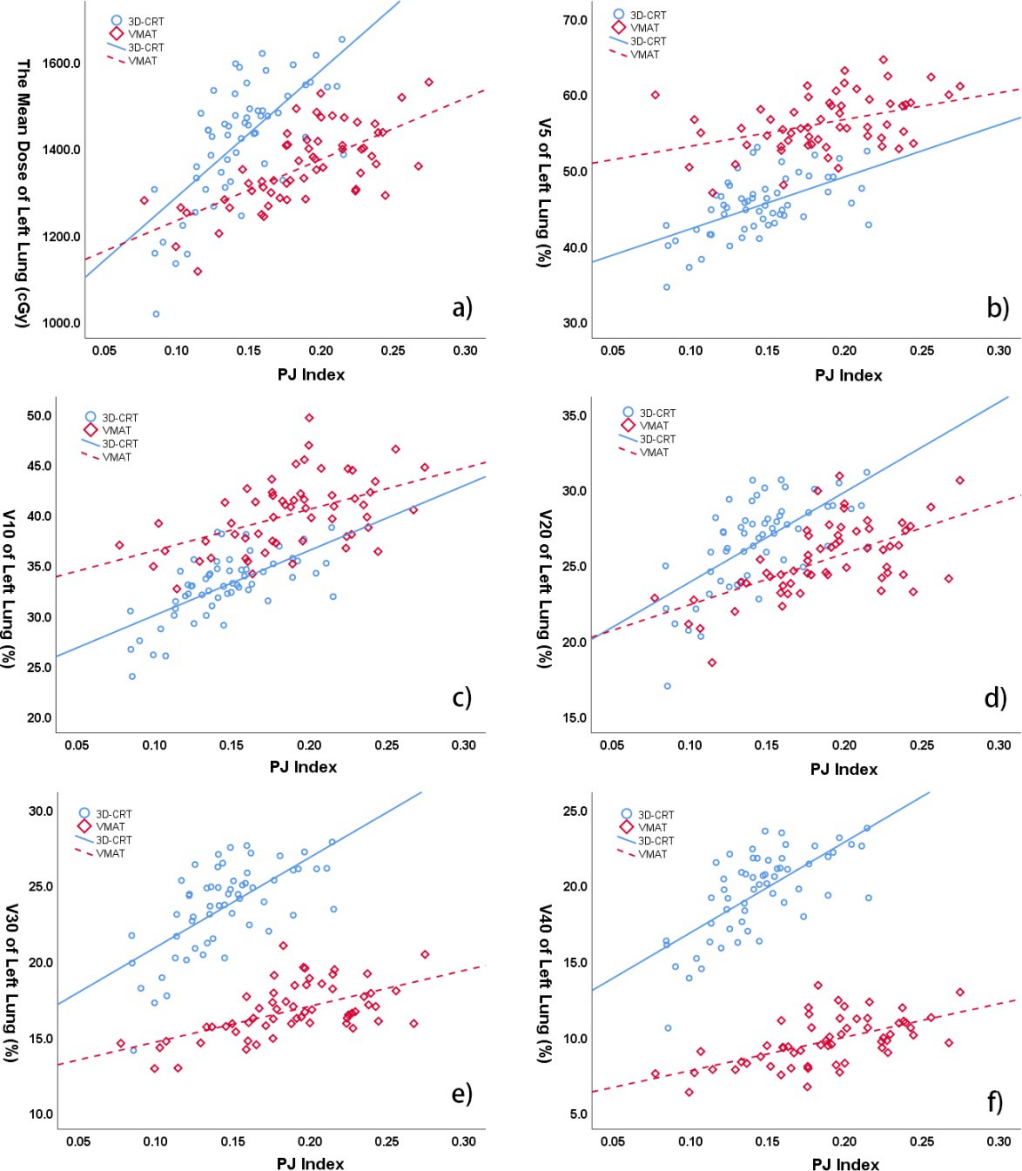
Correlation between PJ index and dose metrics of left lung in 3D-CRT and VMAT. a) PJ index and MLLD (r = 0.697 for 3D-CRT, *p* < 0.01; r = 0.676 for VMAT, *p* < 0.01); b) PJ index and (V_5_ of left lung: r = 0.568 for 3D-CRT, *p* = 0.1; r = 0.407 for VMAT, *p* < 0.01); c) PJ index and V_10_ of left lung (r = 0.663 for 3D-CRT, *p* < 0.01; r = 0.495 for VMAT, *p* < 0.01); d) PJ index and V_20_ of left lung (r = 0.659 for 3D-CRT, *p* < 0.01; r = 0.6 for VMAT, *p* < 0.01); e) PJ index and V_30_ of left lung (r = 0.66 for 3D-CRT, *p* < 0.01; r = 0.578 for VMAT, *p* < 0.01); f) PJ index and V_40_ of left lung (r = 0.691 for 3D-CRT, *p* < 0.01; r = 0.594 for VMAT, *p* < 0.01).

In the VMAT group, PJ index was positively correlated with MLLD (r = 0.676, *p* < 0.01), V_20_ of left lung (r = 0.6, *p* < 0.01), V_30_ of left lung (r = 0.578, *p* 0.01), as well as V_40_ of left lung (r = 0.594, *p* < 0.01) (Fig 3). Similarly, there was statistics significance in the correlation between PJ index and the absorbed dose of left lung in 3D-CRT plans, the correlation was found to be strongly between PJ index and MLLD (r = 0.697, *p* < 0.01), V_5_ (r = 0.568, *p* < 0.01), V_10_ (r = 0.663, *p* < 0.01), V_20_ (r = 0.659, *p* < 0.01), V_30_ (r = 0.66, *p* < 0.01), and V_40_ (r = 0.691, *p* < 0.01) of left lung, respectively (Fig 3).

Linear regression formulas were generated for mean dose of heart and left lung in both 3D-CRT and VMAT plans. The MHD formula of 3D-CRT group was MHD = 4826.59×CJ Index+310.48 (R = 0.857, F = 163.77, P = 0.000), and that of VMAT group was MHD = 1789.29×CJ Index+437.50 (R = 0.45, F = 14.23, P = 0.000). The intersection of the two formulas was that CJ index was 0.042 and MHD was 512.33 cGy. Since the slope of 3D-CRT group was steeper than that of VMAT group, it meant that when CJ index exceeded 4.2%, the MHD of 3D-CRT group would be greater than that of VMAT group. The MLLD formula of 3D-CRT group was MLLD = 2879.54×PJ Index+999.79 (R = 0.697, F = 55.86, P = 0.000), and that of VMAT group was MLLD = 1411.79×PJ Index+1091.88 (R = 0.676, F = 47.11, P = 0.000), and their intersection was PJ index equalled to 0.063 and MLLD equalled to 1180.46 cGy. For the slope of 3D-CRT group was steeper than that of VMAT group, it meant that when PJ index exceeded 6.3%, the MLLD of 3D-CRT group would be greater than that of VMAT group. Furthermore, the average MLLD of 3D-CRT group was 1420.31 cGy, and its corresponding PJ index was 0.146, suggesting that when PJ index was greater than 14.6%, the MLLD of 3D-CRT plan would much greater than that of VMAT plan.

## Discussion

In this study, we identified two geometric indices of CT images of patients with left-sided breast cancer after modified radical mastectomy, and studied the relationship between these metrics and absorbed dose of heart and left lung, including the mean dose of heart and left lung, V_5_, V_30_ of heart and V_5_, V_10_, V_20_, V_30_, V_40_ of left lung. To the best of our knowledge, this is the first study to evaluate these geometric indices and their relationship with the heart and left lung dose.

The results showed that CJ index was significantly correlated with cardiac dose metrics in 3D-CRT group. The correlation coefficients of MHD, V_5_, and V_30_ were 0.857 (*p* < 0.01), 0.814 (*p* < 0.01), and 0.869 (*p* < 0.01), respectively. Recently, Cao et al. indicated in their study that there was a positive linear correlation between cardiac contact distance (CCD_ps_) and MHD (r = 0.63, *p* < 0.01) [19]. They also suggested that there was a negative correlation between the heart-to-chest distance (HCD) and MHD (r = -0.65, *p* < 0.01). Similarly, Mendez et al. investigated other predictors (4^th^ Arch and 5^th^ Arch) in another study. 4^th^ Arch and 5^th^ Arch were line segment from the left edge of sternum to the anterior part of left lung parenchyma at the level of fourth or fifth costal arch [18]. Their study showed that the correlation coefficient between 4^th^ Arch and MHD was 0.61 (*p* < 0.05), and that between 4^th^ Arch and V_25_ of heart was 0.57 (*p* < 0.05). Despite its reasonable prediction capacity, it was not clear whether the CT scan can accurately obtain the slice of the 4^th^ costal arch because the thickness of the 4^th^ costal arch is far beyond the range of CT scans. In our research, the geometric metrics covered the entire range of organs at risk, so we could ignore the thickness of CT slice. Other studies had also shown that the maximum distance from the heart to the chest wall was related to MHD [20–22], and could reliably estimate the cardiac exposure of patients receiving breast RT. Consistent with previous studies, our results implied that the dose distribution of the heart depends largely on the proximity of the heart to the radiation field. However, in the VMAT group, we only observed a moderate correlation between CJ index and cardiac dosimetry. We hypothesized that because the radiation field of VMAT technique was arc-shaped, it would deliver the dose to the heart in a nonlinear way, thus the correlation in VMAT group was weaker compared to 3D-CRT group.

Interestingly, the MHD value at the intersection of the two MHD formulas in this study was slightly less than MHD in both groups. Considering that the slope of MHD formulas in 3D-CRT group was greater than that in VMAT group, when CJ index was larger than 4.2%, MHD in 3D-CRT group would surpass the MHD in VMAT group (Fig 2). Therefore, we recommend VMAT plan when CJ index is greater than 4.2%. When the CJ index is less than 4.2%, 3D-CRT plan should be the first consideration.

In this study, we also examined the effect of PJ index on dose metrics of left lung. There was a strong positive correlation between PJ index and absorbed dose parameters in 3D-CRT and VMAT plans, as illustrated in Fig 3. Although the intersection of MLLD formulas was PJ index equalled to 0.063 and MLLD equalled to 1180.46 cGy, we did not think it has meaningful clinical significance, because the PJ index would always be more than 6.3% in breast cancer with supraclavicular region. Since the slops of MLLD formula in 3D-CRT plans was steeper than that in VMAT plans, with the increase of PJ index, the absorbed dose of left lung in 3D-CRT group changed more than that in VMAT group. In addition, the average MLLD of 3D-CRT group was 1420.31 cGy, and the corresponding PJ index was 14.6%. Therefore, we suggest that when the PJ index is greater than 14.6%, VMAT plan could be preferred, otherwise 3D-CRT plan could be selected.

In this study, we also found that compared with 3D-CRT plan, VMAT plan exhibited superior dosimetric advantages at high-doses. The V_30_ of heart and V_30_, V_40_ of the ipsilateral lung were considerably lower in VMAT plans than in 3D-CRT plans (*p* < 0.01). Moreover, the MLLD of ipsilateral lung in VMAT group were less than that in 3D-CRT group, which was consistent with the study of Liu et al. and Mo et al [14, 15], but contrary to the study of Bogue [13]. By the way, we discovered that the PJ index was larger in the VMAT group than in the 3D-CRT group. Although we observed similar MHD in the two groups in this study, the larger PJ index in the VMAT group indicated that VMAT plan plays an important role in reducing cardiac doses. However, the low-dose radiation area of VMAT group was larger than that of 3D-CRT group, which was consistent with other studies [13–15].

There are several limitations in this study. First, we recognized that the prediction of heart and lung dose was sophisticated, and it may not be perfect to use only geometric indices. Second, there was no standard VMAT, and the dose in OAR depend on widely varying technology, beam setup, OAR constraints, etc. Thus, the relation between geometric indices and dose distributions may lack of universality. Third, although CJ index was similar among groups, it should be noted that the PJ index was different among the groups, which indicated that the cases in the two groups were not completely matched, the VMAT group may have larger or longer target area.

## Conclusion

We propose two geometric indices which were found to be associated with the dose distribution in the heart and left lung. These indices are easily implementable and low-cost and requiring little time commitment in clinical treatment planning process. The implement of these indices would aid the decision making for the choice of 3D-CRT or VMAT plan in the clinical process. We recommend that VMAT plan is preferable when CJ index is greater than 4.2% and/or PJ index is greater than 14.6%, otherwise 3D-CRT plan should be used. Since this study was a retrospective analysis, our hypothesis require validation in the future prospective randomized research.

## Supporting information

S1 Fig BA plot of cardiac dose. (TIF)

S2 Fig BA plot of pulmonary dose. (TIF)

S1 File All data. (XLSX)

## References

[1] Wang H, Kong L, Zhang C, Chen D, Zhu H, Yu J. Should all breast cancer patients with four or more positive lymph nodes who underwent modified radical mastectomy be treated with postoperative radiotherapy? A population-based study. Oncotarget 2016, 7(46):75492–75502. doi: 10.18632/oncotarget.12260 27690343 PMC5342755

[2] Stecklein SR, Shen X, Mitchell MP: Post-Mastectomy Radiation Therapy for Invasive Lobular Carcinoma: A Comparative Utilization and Outcomes Study. Clin Breast Cancer 2016, 16(4):319–326. doi: 10.1016/j.clbc.2016.02.001 26943990

[3] Rusthoven CG, Rabinovitch RA, Jones BL, Koshy M, Amini A, Yeh N, et al. The impact of postmastectomy and regional nodal radiation after neoadjuvant chemotherapy for clinically lymph node-positive breast cancer: a National Cancer Database (NCDB) analysis. Ann Oncol 2016, 27(5):818–827. doi: 10.1093/annonc/mdw046 26861597

[4] Darby S, McGale P, Correa C, Taylor C, Arriagada R, Clarke M, et al. Effect of radiotherapy after breast-conserving surgery on 10-year recurrence and 15-year breast cancer death: meta-analysis of individual patient data for 10,801 women in 17 randomised trials. Lancet 2011, 378(9804):1707–1716. doi: 10.1016/S0140-6736(11)61629-2 22019144 PMC3254252

[5] McGale P, Taylor C, Correa C, Cutter D, Duane F, Ewertz M, et al. Effect of radiotherapy after mastectomy and axillary surgery on 10-year recurrence and 20-year breast cancer mortality: meta-analysis of individual patient data for 8135 women in 22 randomised trials. Lancet 2014, 383(9935):2127–2135. doi: 10.1016/S0140-6736(14)60488-8 24656685 PMC5015598

[6] Clarke M, Collins R, Darby S, Davies C, Elphinstone P, Evans V, et al. Effects of radiotherapy and of differences in the extent of surgery for early breast cancer on local recurrence and 15-year survival: an overview of the randomised trials. Lancet 2005, 366(9503):2087–2106. doi: 10.1016/S0140-6736(05)67887-7 16360786

[7] Demirci S, Nam J, Hubbs JL, Nguyen T, Marks LB: Radiation-induced cardiac toxicity after therapy for breast cancer: interaction between treatment era and follow-up duration. Int J Radiat Oncol Biol Phys 2009, 73(4):980–987. doi: 10.1016/j.ijrobp.2008.11.016 19251085

[8] Darby SC, Ewertz M, McGale P, Bennet AM, Blom-Goldman U, Bronnum D, et al. Risk of ischemic heart disease in women after radiotherapy for breast cancer. N Engl J Med 2013, 368(11):987–998. doi: 10.1056/NEJMoa1209825 23484825

[9] van den Bogaard VA, Ta BD, van der Schaaf A, Bouma AB, Middag AM, Bantema-Joppe EJ, et al. Validation and Modification of a Prediction Model for Acute Cardiac Events in Patients With Breast Cancer Treated With Radiotherapy Based on Three-Dimensional Dose Distributions to Cardiac Substructures. J Clin Oncol 2017, 35(11):1171–1178. doi: 10.1200/JCO.2016.69.8480 28095159 PMC5455600

[10] Jeba J, Isiah R, Subhashini J, Backianathan S, Thangakunam B, Christopher DJ: Radiation Pneumonitis After Conventional Radiotherapy For Breast Cancer: A Prospective Study. J Clin Diagn Res 2015, 9(7):XC01–XC05. doi: 10.7860/JCDR/2015/13969.6211 26393189 PMC4573021

[11] Wen G, Tan YT, Lan XW, He ZC, Huang JH, Shi JT, et al. New Clinical Features and Dosimetric Predictor Identification for Symptomatic Radiation Pneumonitis after Tangential Irradiation in Breast Cancer Patients. J Cancer 2017, 8(18):3795–3802. doi: 10.7150/jca.21158 29151967 PMC5688933

[12] Grantzau T, Overgaard J: Risk of second non-breast cancer among patients treated with and without postoperative radiotherapy for primary breast cancer: A systematic review and meta-analysis of population-based studies including 522,739 patients. Radiother Oncol 2016, 121(3):402–413. doi: 10.1016/j.radonc.2016.08.017 27639892

[13] Bogue J, Wan J, Lavey RS, Parsai EI: Dosimetric comparison of VMAT with integrated skin flash to 3D field-in-field tangents for left breast irradiation. J Appl Clin Med Phys 2019, 20(2):24–29. doi: 10.1002/acm2.12527 30653831 PMC6371015

[14] Mo JC, Huang J, Gu WD, Gao M, Ning ZH, Mu JM, et al. A dosimetric comparison of double-arc volumetric arc therapy, step-shoot intensity modulated radiotherapy and 3D-CRT for left-sided breast cancer radiotherapy after breast-conserving surgery. Technol Health Care 2017, 25(5):851–858. doi: 10.3233/THC-160746 29103057

[15] Liu H, Chen X, He Z, Li J: Evaluation of 3D-CRT, IMRT and VMAT radiotherapy plans for left breast cancer based on clinical dosimetric study. Comput Med Imaging Graph 2016, 54:1–5. doi: 10.1016/j.compmedimag.2016.10.001 27838084

[16] Ashby O, Bridge P: Late effects arising from volumetric modulated arc therapy to the breast: A systematic review. Radiography (Lond) 2020. doi: 10.1016/j.radi.2020.08.003 32819824

[17] Lazzari G, Terlizzi A, Leo MG, Silvano G: VMAT radiation-induced nausea and vomiting in adjuvant breast cancer radiotherapy: The incidental effect of low-dose bath exposure. Clin Transl Radiat Oncol 2017, 7:43–48. doi: 10.1016/j.ctro.2017.09.009 29594228 PMC5862677

[18] Mendez LC, Louie AV, Moreno C, Wronski M, Warner A, Leung E, et al. Evaluation of a new predictor of heart and left anterior descending artery dose in patients treated with adjuvant radiotherapy to the left breast. Radiat Oncol 2018, 13(1):124. doi: 10.1186/s13014-018-1069-z 29973243 PMC6032565

[19] Cao N, Kalet AM, Young LA, Fang LC, Kim JN, Mayr NA, et al. Predictors of cardiac and lung dose sparing in DIBH for left breast treatment. Phys Med 2019, 67:27–33. doi: 10.1016/j.ejmp.2019.09.240 31629280

[20] Coon AB, Dickler A, Kirk MC, Liao Y, Shah AP, Strauss JB, et al. Tomotherapy and multifield intensity-modulated radiotherapy planning reduce cardiac doses in left-sided breast cancer patients with unfavorable cardiac anatomy. Int J Radiat Oncol Biol Phys 2010, 78(1):104–110. doi: 10.1016/j.ijrobp.2009.07.1705 20004529

[21] Taylor CW, McGale P, Povall JM, Thomas E, Kumar S, Dodwell D, et al. Estimating cardiac exposure from breast cancer radiotherapy in clinical practice. Int J Radiat Oncol Biol Phys 2009, 73(4):1061–1068. doi: 10.1016/j.ijrobp.2008.05.066 18973978

[22] Hiatt JR, Evans SB, Price LL, Cardarelli GA, Dipetrillo TA, Wazer DE: Dose-modeling study to compare external beam techniques from protocol NSABP B-39/RTOG 0413 for patients with highly unfavorable cardiac anatomy. Int J Radiat Oncol Biol Phys 2006, 65(5):1368–1374. doi: 10.1016/j.ijrobp.2006.03.060 16863924

