# Supplementary figures and images for: Predictors of heart and lung dose in left-sided breast cancer treated with VMAT relative to 3D-CRT: A retrospective study
Source: PLoS One. 2021 Jun 9;16(6):e0252552. doi: 10.1371/journal.pone.0252552 (PMC8189517; doi:10.1371/journal.pone.0252552)

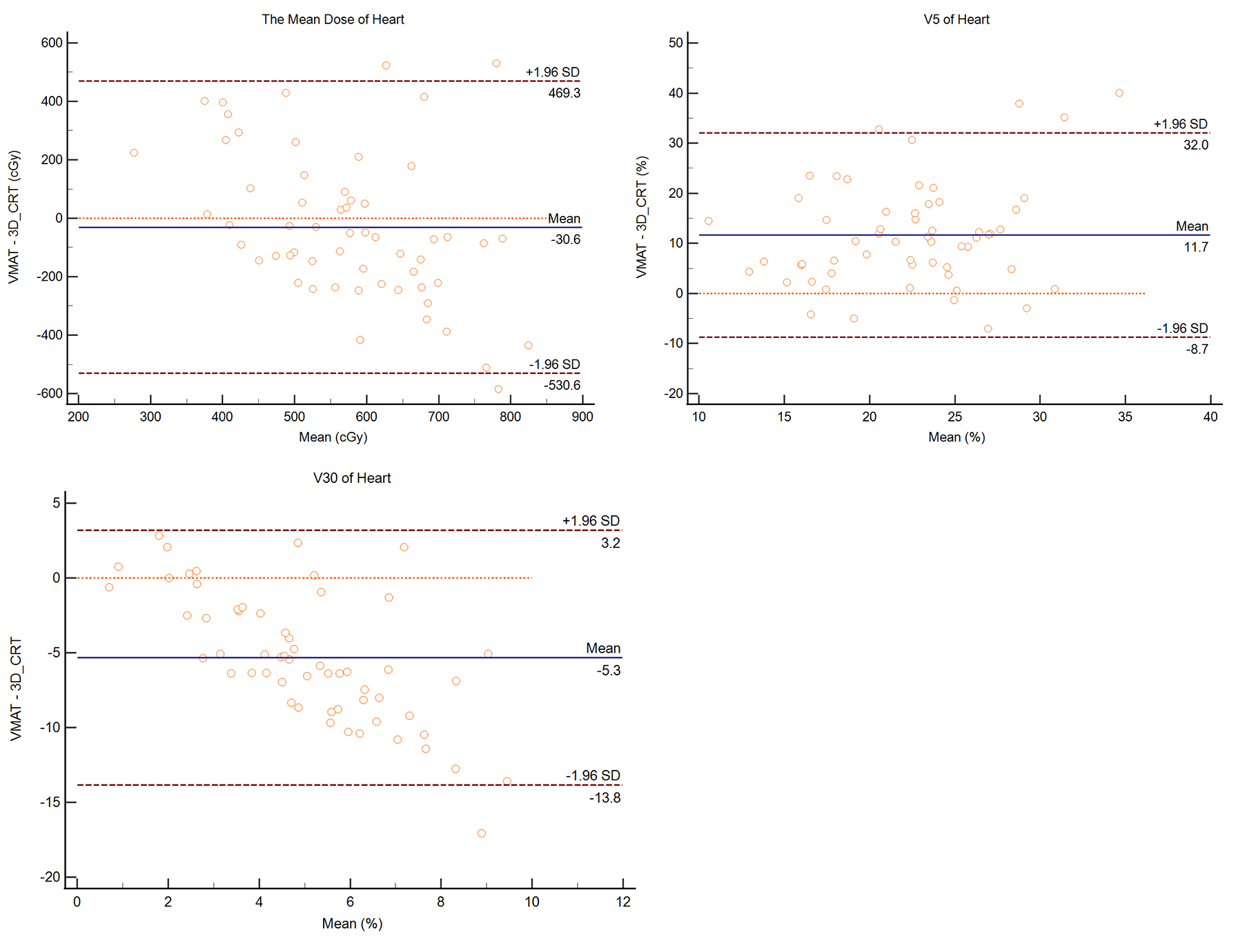

Supplement: S1 Fig — (TIF) [file pone.0252552.s001.tif]

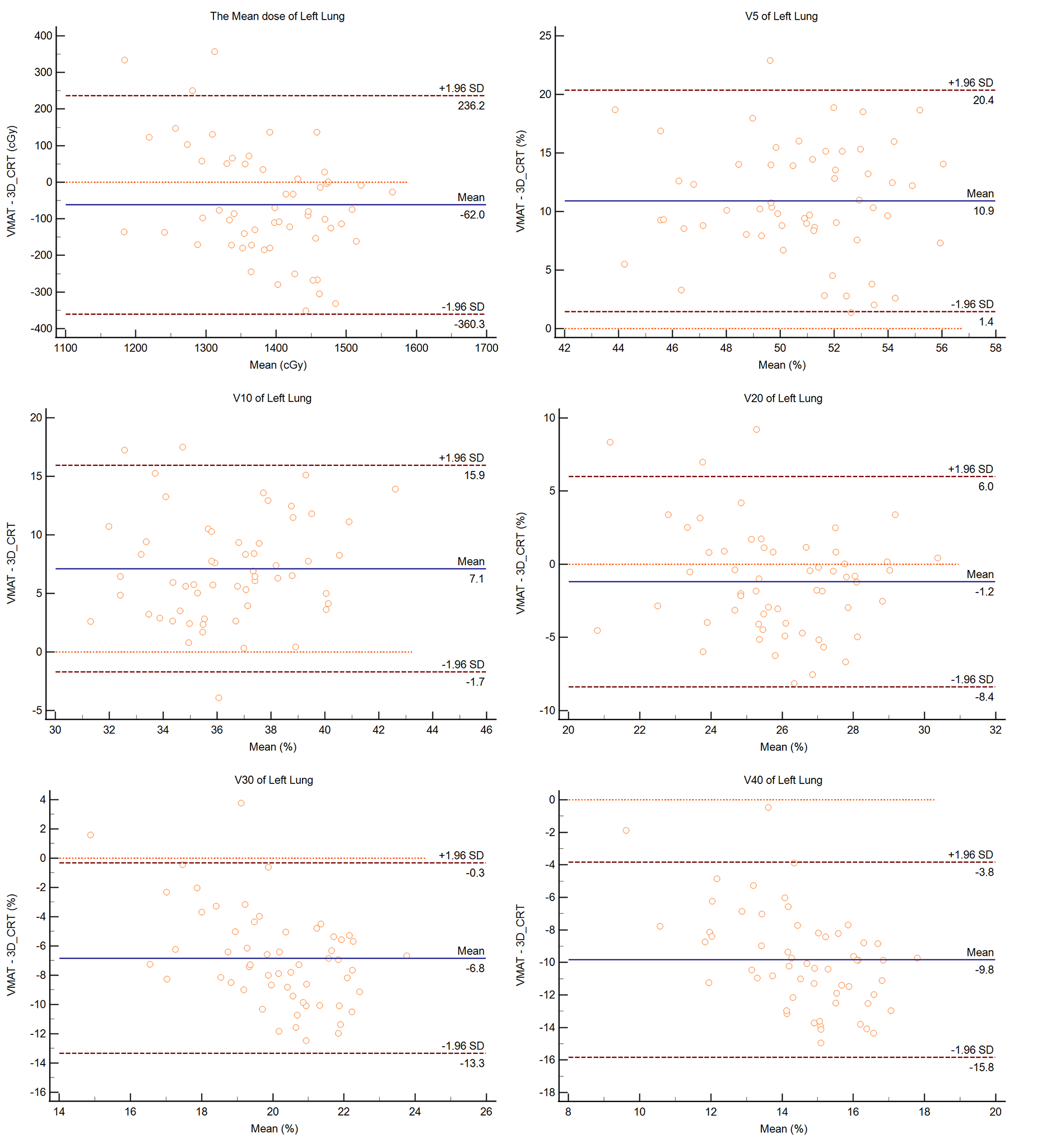

Supplement: S2 Fig — (TIF) [file pone.0252552.s002.tif]
